# Warm Springs, Early Lay Dates, and Double Brooding in a North American Migratory Songbird, the Black-Throated Blue Warbler

**DOI:** 10.1371/journal.pone.0059467

**Published:** 2013-04-02

**Authors:** Andrea K. Townsend, T. Scott Sillett, Nina K. Lany, Sara A. Kaiser, Nicholas L. Rodenhouse, Michael S. Webster, Richard T. Holmes

**Affiliations:** 1 Cornell Lab of Ornithology & Department of Neurobiology & Behavior, Cornell University, Ithaca, New York, United States of America; 2 Migratory Bird Center, Smithsonian Conservation Biology Institute, National Zoological Park, Washington, District of Columbia, United States of America; 3 Department of Biological Sciences, Dartmouth College, Hanover, New Hampshire, United States of America; 4 Department of Biological Sciences, Wellesley College, Wellesley, Massachusetts, United States of America; Université de Sherbrooke, Canada

## Abstract

Numerous studies have correlated the advancement of lay date in birds with warming climate trends, yet the fitness effects associated with this phenological response have been examined in only a small number of species. Most of these species–primarily insectivorous cavity nesters in Europe–exhibit fitness declines associated with increasing asynchrony with prey. Here, we use 25 years of demographic data, collected from 1986 to 2010, to examine the effects of spring temperature on breeding initiation date, double brooding, and annual fecundity in a Nearctic - Neotropical migratory songbird, the black-throated blue warbler (Setophaga caerulescens). Data were collected from birds breeding at the Hubbard Brook Experimental Forest, New Hampshire, USA, where long-term trends toward warmer springs have been recorded. We found that black-throated blue warblers initiated breeding earlier in warmer springs, that early breeders were more likely to attempt a second brood than those starting later in the season, and that double brooding and lay date were linked to higher annual fecundity. Accordingly, we found selection favored earlier breeding in most years. However, in contrast to studies of several other long-distance migratory species in Europe, this selection pressure was not stronger in warmer springs, indicating that these warblers were able to adjust mean lay date appropriately to substantial inter-annual variation in spring temperature. Our results suggest that this North American migratory songbird might not experience the same fecundity declines as songbirds that are unable to adjust their timing of breeding in pace with spring temperatures.

## Introduction

Changing climate has been linked to changes in phenology or distribution in more than half of nearly 1600 marine, freshwater, and terrestrial species studied [1]. Advancing lay date of birds in warmer springs is one of the most widely reported responses [2], [3]. Indeed, a recent review [4] documented that 75% of species studied laid eggs earlier in warmer years, and 59% have advanced their mean lay date over time. Very little is known, however, about the fitness consequences of such phenological changes for most of these species, which limits our ability to draw inferences about the effects of warming climate trends on avian population dynamics and trends.

The fitness effects of earlier breeding vary among the species of birds that have been studied. In a population of year-round resident song sparrows (*Melospiza melodia*) in North America, for example, birds initiated breeding earlier in warm springs, and had higher reproductive success when breeding began earlier [5]. In contrast, among tits (*Paridae*) and flycatchers (*Muscicapidae*) in Europe, breeding success generally declines in warm springs, when timing of peak offspring energy demands mismatches with peak prey abundance [6], [7]. Based primarily on data from the great tit (Parus major) and pied flycatcher (Ficedula hypoleuca), some authors have suggested that these phenological mismatches and associated fitness declines might become more pronounced as global climate continues to change [8], particularly for insectivores specializing on prey with a narrow period of peak abundance [7].

Migratory birds with relatively fixed cues for spring migration (e.g., photoperiod) comprise one group that might have particular difficulty matching breeding phenology with changing climatic conditions [9]–[11]. The capacity of pied flycatchers to advance their lay date to match advancement of peak prey abundance, for example, appears to be constrained by day-length cues that determine departure from their tropical winter quarters, leading to intensified selection pressure for earlier lay date: early-breeding birds have increasingly higher reproductive success than later-breeding birds [12]. Likewise, a recent study of six woodland songbird species in the UK suggested that selection pressure for earlier breeding is intensifying among migratory (but not resident) birds [13]. Overall, avian responses to climate change appear to be slower or more constrained for long-distance migrants than for residents and short-distance migrants [14], which might contribute to population declines among long-distance migratory species [15], [16].

Not all insectivorous birds, however, might experience fitness or population declines in response to warming climate trends. Differential responses could arise from differences in life history or ecology. The relationship between fitness and the timing breeding, for example, might differ between species that produce only one brood (single brooders) or more than one brood (double brooders) per breeding season [5], [17]–[20], or might depend on the timing and duration of peaks in food abundance [21], [22]. Additional demographic data are needed to test hypotheses about how climate change will affect avian fitness.

Here, we used a long-term dataset to examine the effects of spring temperature on breeding initiation date, double brooding, annual fecundity, and selection pressure for early breeding in the black-throated blue warbler (Parulidae: Setophaga caerulescens), a Nearctic – Neotropical migratory passerine. The study was conducted over a 25-year period in the Hubbard Brook Experimental Forest, New Hampshire, United States of America [23], where temperatures have increased [24] and spring leaf phenology has advanced [25] between 1957 and 2004. We used temperature and warbler demographic data from this site to address the following questions:

- Does variation in temperature affect mean first lay date?
- How do first lay date and temperature affect the probability of double brooding and annual fecundity?
- Does selection pressure to breed earlier intensify in warm years?
- What are the implications of these findings for how double brooded migratory passerines could respond to projected climate warming?

We emphasize mean lay date because arrival date might be constrained by relatively fixed migratory cues [9] or by events occurring in non-breeding areas [26], [27]. Mean lay date at the population level could advance, despite constrained arrival date, if birds have flexibility in the timing of breeding after arrival [12]. Furthermore, detection of mean first lay date is likely to be less sensitive to fluctuations in population density or observer bias than detection of first occurrences [10], [28]. Because older black-throated blue warblers tend to arrive and settle earlier on breeding grounds than do yearlings [29], [30], we also examined the effects of breeder age class on timing of breeding and annual fecundity.

## Methods

### Study System and Field Methods

Black-throated blue warblers breed in mature, northern hardwood forests of eastern North America and winter in the Greater Antilles. Lepidoptera larvae represent the majority of the prey biomass fed to the offspring of this insectivorous warbler, with the balance comprising flying Diptera, Hymenoptera and adult Lepidoptera, as well as Coleoptera and Arachnida [30]–[32]. Black-throated blue warblers are sexually dichromatic, and males defend exclusive, non-overlapping territories during the breeding season. Females build open-cup nests in understory vegetation and incubate eggs; both sexes feed nestlings and fledglings.

Our research was conducted in the 3160-ha Hubbard Brook Experimental Forest (Woodstock, New Hampshire, USA), a mature, unfragmented, second-growth hardwood forest embedded within the 317,478-ha White Mountain National Forest [23]. We used data from a 60-ha study plot located within an elevational band of 450–600 m ASL, where nest initiation and fecundity data were collected from 1986 through 2010. Dominant canopy trees included sugar maple (Acer saccharum), American beech (Fagus grandifolia), and yellow birch (Betula alleghaniensis), with red spruce (Picea rubens) and balsam fir (Abies balsamea). The patchy understory was composed of hobblebush (Viburnum alnifolium) and striped maple (A. pensylvanicum), as well as saplings of canopy species. Abundance of black-throated blue warblers in this forest has been stable since at least 1969 [33], [34].

Adults were captured in mist nets, given unique combinations of colored leg bands and a numbered, aluminium USGS leg band, and aged as either yearlings or older breeders based on plumage characters [29]. We located and monitored nesting attempts (successful and unsuccessful) of all warblers within the study area. Black-throated blue warblers at Hubbard Brook often attempt multiple broods per season, either as renests (i.e., attempts to produce another brood following a nest failure) or double broods (i.e., attempts to produce another brood after successfully fledging offspring). Although they are usually socially monogamous, approximately 10% of males are bigamous (i.e., associated with two nesting females, either sequentially or simultaneously) each year [35]. We focused on monogamous pairs in these analyses because first lay date and fecundity of females paired to bigamous males are likely to be influenced by social factors, such as interactions between females, and because second females often appear on male territories in the middle of the breeding season.

### Ethics Statement

This work was performed under protocols approved by the Institutional Animal Care and Use Committees of Dartmouth College, Wellesley College, the Smithsonian National Zoological Park, and Cornell University.

### Datasets and Statistical Methods

We used temperature data from a U.S. Forest Service weather station located within 1 km of and within the same elevational band as our study plot (490 m ASL), at which daily mean temperatures have been recorded since 1956. We defined spring temperature as the annual mean of mean daily temperatures recorded from 15 March through 18 May (Table 1). This start point (15 March) is relevant to warbler breeding initiation because temperature accumulation after 15 March has been linked to the advancement of spring leaf phenology [25] and the development potential in Lepidoptera [24] at Hubbard Brook. This endpoint (18 May) is relevant to warbler breeding initiation because most birds have settled on territories by 15–20 May, with nest initiation beginning 3–7 days after arrival, depending on weather conditions [30]. We examined temporal changes in temperature in linear regression models with spring temperature as the response and year as the predictor. For this and other models in which we looked for a trend over time, we first carried out preliminary analyses to test the assumption of no temporal autocorrelation across years. Following Zuur et al. (2009) [36], we regressed mean annual values of each response variable (temperature, start date, and standardized selection differentials (SSD)) against year in ordinary linear regression models (function “gls” in R library “nlme”). We re-ran each model with (1) an auto-regressive model of order 1 (AR-1) auto-correlation structure, specifying the “corAR1” correlation option with respect to year, and (2) a compound symmetry auto-correlation structure, specifying the “corCompSymm” correlation option with respect to year. We compared the models with and without auto-correlation structure using Akaike’s Information Criterion (AIC). Models with ΔAIC values <2 were considered to have equal support given the data. We found no evidence that including either auto-correlation structure improved fit of models with temperature or start date as the response variable (ΔAIC <2), and we did not incorporate them into subsequent models. However, the AR-1 auto-correlation structure did improve the fit of the model with mean SSD as the response variable (ΔAIC >2), and we used this structure in subsequent analyses of SSD. All statistics were carried out in R v. 2.11.1 [37]. Parameter estimates (β) are given as means ±1 SE.

**Table 1:** Spring temperature and sample size of breeding warbler pairs in each year.

Year	Spring temperature (°C)	# breeding pairs
1986	6.63	8
1987	6.32	5
1988	4.95	10
1989	4.17	10
1990	5.40	10
1991	6.85	15
1992	3.52	11
1993	6.12	7
1994	4.37	10
1995	3.80	9
1996	4.46	5
1997	2.46	9
1998	7.66	9
1999	6.38	14
2000	6.15	13
2001	6.11	23
2002	4.22	15
2003	4.45	14
2004	5.63	12
2005	5.11	20
2006	5.85	29
2007	4.94	16
2008	5.18	14
2009	6.52	16
2010	8.31	5

Spring temperatures were the average of daily means from 15 March through 18 May each year, recorded at the Hubbard Brook Experimental Forest, New Hampshire.

For analyses of individual start date and annual fecundity, we included only pairs for which we had complete reproductive information for a given year (n = 309 pair-years; distribution of pair-years across years shown in Table 1). These pair-years were associated with 234 males, 263 females, and 297 pairs because some birds bred in multiple seasons. For analyses involving first lay date, we included initiation date of only the first clutch produced in each pair-year. We excluded data from pair-years for which first recorded clutch was initiated more than 24 days (the length of a nesting cycle) after the first clutch initiated in that year because these likely represented cases in which we missed their initial nesting attempt of the season. Similar cut-off dates have been used in other studies of first lay date [38]. Patterns of lay date and fecundity with spring temperature were comparable without this 24-day filter (data not shown). We used total number of young fledged from all clutches produced in each of the pair-years as the measure of annual fecundity. For analyses of clutch size with lay date, we considered only first clutches per territory because clutch size tends to be smaller for second clutches and renests [30].

We assessed the effect of variation in temperature on mean first lay date in a linear mixed effects model (LME) fit by restricted maximum likelihood (REML) (R extension package “nlme”), specifying first lay date as the response and spring temperature, year, and breeder age class as fixed effects. We included breeder age class because prior studies indicate that both male and female age class can affect clutch initiation date [29], [30]. Pairs were categorized as (1) yearling males and females; (2) yearling males and older females; (3) older males and yearling females; and (4) older males and females. To account for non-independence of responses within years and for repeated measures of individual birds across years, we examined the full model with year (here specified as a categorical factor), male breeder, female breeder, or breeding pair as varying-intercept random effects, and then compared these models to a model without random effects using AIC [36]. We found support for an effect of year (ΔAIC >2), but no support for an effect of breeder identity (ΔAIC <2), and therefore included only year as a random categorical effect in subsequent models. We then determined optimal fixed structure of covariates by sequentially dropping the least significant term (likelihood ratio test; α = 0.05 level) and refitting the model [36]. Significant probability values were derived from the optimal model, whereas values for non-significant terms were derived by individually reintroducing each non-significant term into the optimal model.

We examined relationships among first lay date, temperature, double brooding, and fecundity using generalized linear mixed models (GLMERs) fit by the Laplace approximation (R extension package “lme4” [39]). First, we examined the effects of first lay date and breeder pair age class on: the probability of double brooding after successfully fledging a brood (0/1; binomial distribution), annual fecundity (Poisson distribution), and size of first clutch (Poisson distribution). Second, to examine how spring temperature directly affected warbler fecundity, we specified spring temperature as a predictor instead of lay date in each of these models. Finally, we tested for effects of double brooding and breeder age class on annual fecundity (Poisson distribution). Parameter estimates from GLMERs with binomial and Poisson distributions were given in the logit and log scale, respectively. As before, year (specified as a categorical factor) was retained as a random effect in the final models (ΔAIC >2), but breeder identity was not because it did not improve model fit (ΔAIC <2).

We calculated standardized selection differentials (SSDs) to quantify the strength of selection for an earlier lay date; SSD = (LD_w_ – LD)/SD), where LD = mean annual first lay date, LD_w_ = mean annual first lay date, weighted by the number of offspring fledged per territory, and SD = annual standard deviation of first lay date. Negative differentials suggest selection for early laying, with strength of selection increasing with distance from zero, whereas positive differentials suggest selection for later breeding [13]. We used number of offspring fledged per territory per year as our index of reproductive success, rather than number of breeding recruits in the next breeding season, because return of yearlings to their natal site is extremely low [30]. We examined temperature effects on selection pressure using in an ordinary linear regression model with selection differential as the response and year and mean spring temperature as predictors. We specified the “corAR1” correlation option with respect to year to account for non-independence of residuals among years.

## Results

### Variation in Temperature and First Lay Date

**Figure 1:**
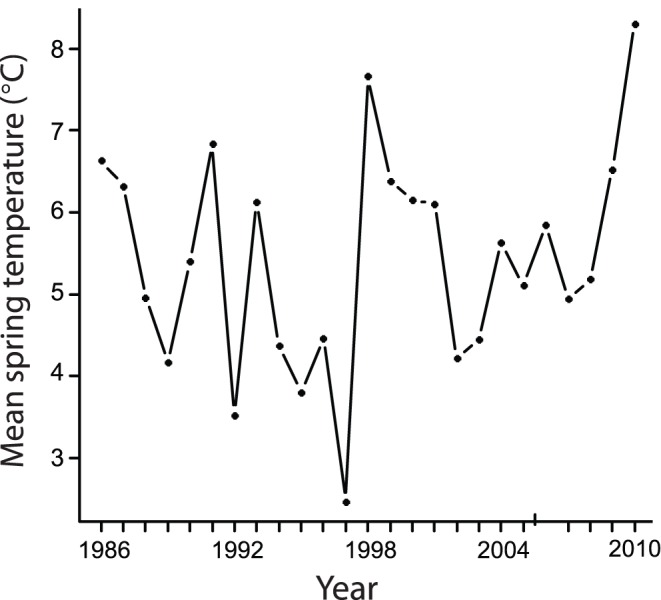
Variation in mean spring temperature over the 25 years of this demographic study (1986–2010). Mean spring temperature (from 15 March –18 May) varied 3.3-fold across years, but did not increase significantly over this 25-year time period at the Hubbard Brook Experimental Forest, New Hampshire, USA.

Mean spring temperatures at Hubbard Brook were highly variable between 1986 and 2010 (Table 1), ranging from 2.46 to 8.31°C (mean = 5.4±1.3°C), but did not show a significant directional change over the time period of the demographic study (1986–2010; β (year) = 0.03±0.04°C/year; t_23_ = 1.3; p = 0.41; Fig. 1). Likewise, we found no advancement in mean first lay date over the period of the demographic study (1986–2010; β (year) = 0.07±0.05 days/year; t_307_ = 1.44; p = 0.15). Temperatures did, however, get warmer over a longer time series: mean spring temperature increased by approximately 2.1°C between 1956 (when weather monitoring at Hubbard Brook first began) and 2010 (β (year) = 0.03±0.01°C/year; t_53_ = 0.10.4; p = 0.002).

Black-throated blue warblers began breeding earlier in warmer springs (with breeder age held constant; Table 2). Pairs containing yearling males bred significantly later than did pairs of older birds (with spring temperature held constant; Table 2). Mean first clutch date across all years was 30–31 May (range: 18–19 May to 23–24 June).

**Table 2:** Fixed effects from a linear mixed model* predicting first lay date of black-throated blue warblers.

Term	ß ± SE	DF	T	P
Spring temperature (°C)	−2.27±0.44	23	−5.2	<0.001
Year	0.08±0.08	22	1.1	0.27
Yearling female^a^ vs. older pair	1.43±0.80	281	1.4	0.16
Yearling male^a^ vs. older pair	2.34±0.77	281	3.0	0.003
Yearling pair vs. older pair	4.44±0.72	281	6.2	<0.001

* Year specified as random effect to account for non-independence of measurements within years.

a Mixed-age pair; yearling male paired to older female or yearling female paired to older male.

Spring temperatures were the average of daily means from 15 March through 18 May each year, recorded at the Hubbard Brook Experimental Forest, New Hampshire.

### First Lay Date, Double Brooding, and Annual Fecundity

**Figure 2:**
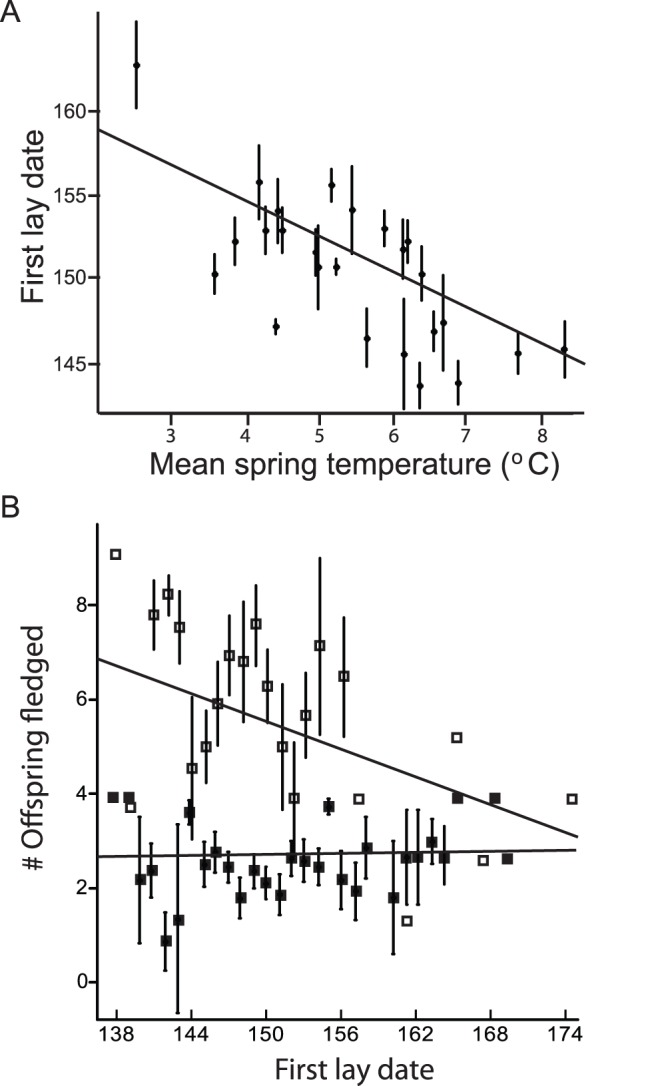
Relationships between spring temperatures, breeding initiation date, and fecundity of black-throated blue warblers. Warblers (a) initiated breeding earlier in warmer springs (β(temperature) = −2.3±0.30; t_307_ = −8.2; p<0.001); and (b) fledged more offspring when breeding initiation date was earlier. Fecundity was higher for birds with an earlier breeding initiation dates among birds that attempted second broods (open squares), but not among single brooders (closed squares; linear model with mean number of offspring fledged as the response, and clutch initiation date, double brooding (yes/no), and the interaction between initiation date and double brooding as predictors (F_3, 48_ = ; p<0.001; R^2^ = 0.68)). Regression lines based on the above models are given here for the purpose of illustration. Results in the text are based on generalized linear mixed models with year as a random effect.

Birds that initiated breeding earlier were more likely to double brood and had higher annual fecundity than birds that bred later in a season (Fig. 2). Double brooding was common: 31% (97/309) of all females laid second clutches after successfully fledging young from the first clutch. The sample size of breeding pairs and percentage that attempted double broods at each first lay-date is given in Table 3. The probability of laying a second clutch declined with increasing first lay date (GLMER: β [lay date] = −0.11±0.03 logit[double brooding], z = −3.6, p = <0.001; observations = 309 pair-years; number of groups = 25 years for this and all subsequent analyses unless otherwise stated), and the mean annual fecundity (3.7±0.1; range = 0–8 offspring) declined with later first lay date (GLMER: β [lay date] = −0.02±0.005 log[offspring], z = −2.9, p = 0.004). The relationship between lay date and annual fecundity appeared to be mediated, at least in part, by the positive relationship between double brooding and fecundity (Fig. 2): significantly more offspring were fledged per territory-year when double broods were attempted (GLMER: β [double brooding] = 0.7±0.06 log[offspring]; z = 11.0, p<0.001). In contrast, first clutches were not larger for birds that initiated breeding earlier (GLMER: β [lay date] = −0.002±0.004 log[eggs], z = −0.6, p = 0.52; n = 361 clutches, groups = 25 years). Pair age class did not affect the probability of double brooding, annual fecundity, or clutch size, and was removed from these models.

**Table 3:** Percentage of pairs that attempted double-broods with first lay date.

First lay date	% attempting second broods
139	50% (2)
140	86% (7)
141	0% (3)
142	25% (12)
143	33% (9)
144	82% (11)
145	50% (8)
146	39% (18)
147	47% (19)
148	30% (20)
149	36% (22)
150	30% (20)
151	32% (25)
152	35% (23)
153	14% (14)
154	27% (22)
155	13% (16)
156	0% (6)
157	33% (9)
158	13% (8)
159	0% (7)
160	0% (7)
161	0% (3)
162	33% (3)
163	0% (2)
165	0% (3)
166	67% (3)
168	100% (1)
169	0% (1)
170	0% (1)
175	100% (1)

Sample size of pairs that initiated breeding at each lay date given in parentheses.

### Temperature, Double Brooding, and Annual Fecundity

We found no evidence that mean spring temperature directly affected warbler double brooding or fecundity. Probability of laying a second clutch did not increase with temperature (GLMER: β [temperature] = 0.03±0.20 logit[double brooding], z = 0.20, p = 0.86). Likewise, annual fecundity did not increase with mean spring temperature (GLMER: β [temperature] = 0.03±0.04 log[offspring], z = 0.76, p = 0.44). Yearling pairs were less likely to attempt a second brood than pairs of older breeders in this model (Table S1).

### Selection for Early Breeding

**Figure 3:**
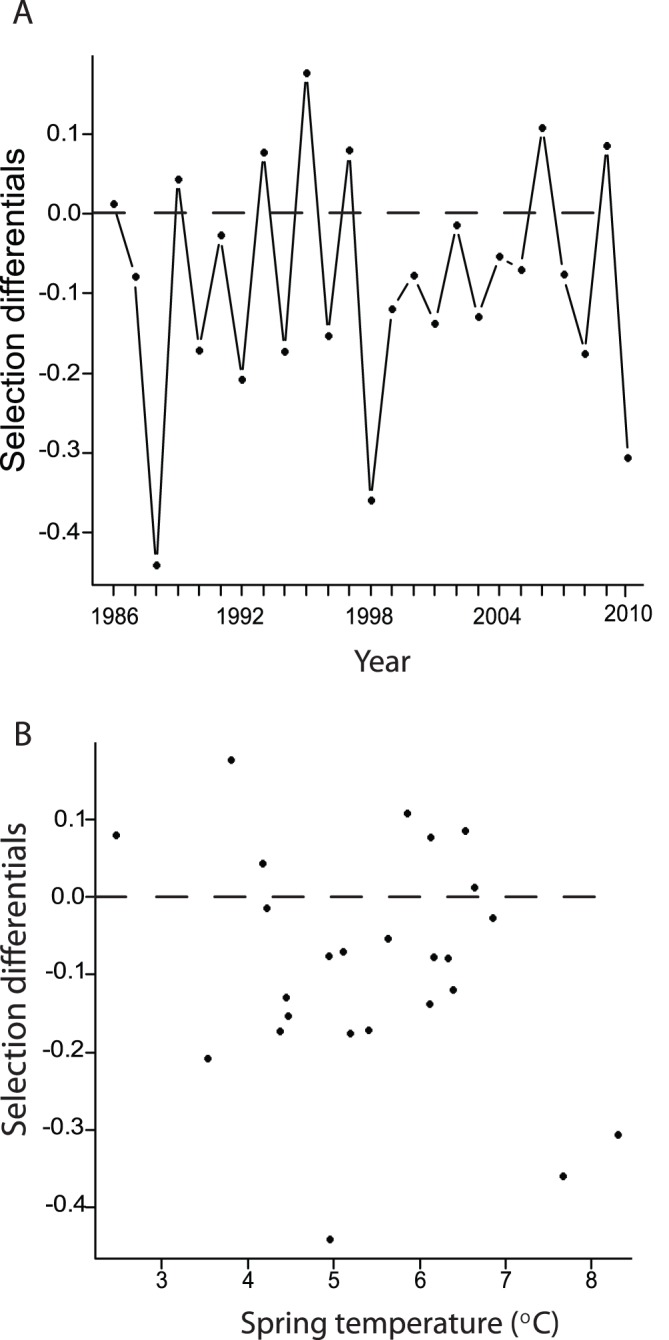
Relationship between selection for earlier breeding (standardized selection differentials), year, and mean spring temperature (15 March–18 May). Points below the zero lines (indicated by dashed lines) indicate selection for earlier breeding. The strength of selection for earlier breeding did not increase (a) over time or (b) with spring temperature. The latter result suggests that black-throated blue warblers were adjusting their lay date sufficiently to the observed variation in spring temperature. Parameter estimates, based on a multiple linear regression model with an AR-1 autocorrelation structure, are given in the text.

Selection favored earlier breeding at Hubbard Brook in most years. Negative selection differentials occurred in 72% (18/25) of years (Fig. 3a) and were significantly more likely to occur than positive differentials (sign test, p<0.04). Moreover, mean annual standardized selection differential across years was negative (−0.09±0.03; range −0.44–0.18), indicating overall selection for earlier breeding. However, in multiple linear regression model with “corAR1” autocorrelation structure, we found no significant effect of year (with temperature held constant: β [year] = 0.00±0.00, t_23_ = 1.3, p = 0.2; Fig. 3a), or spring temperature (with year held constant: β [temperature] = −0.03±0.02, t_23_ = −2.0, p = 0.06; Fig. 3b) on the strength of selection. The correlation between residuals separated by one year (φ) in this model was −0.54, suggesting that the strength of selection on earlier breeding tends to fluctuate from high to low in consecutive years.

## Discussion

We found that black-throated blue warblers at Hubbard Brook initiated breeding earlier in warmer springs. Birds that bred earlier in the season had a fecundity advantage over later-breeding birds: early breeders were more likely to attempt a second brood than birds that started breeding later in the season, and double brooding and lay date were linked to higher annual fecundity. Black-throated blue warblers generally appeared to be under pressure to breed earlier in the season, although selection pressure for early breeding was not significantly stronger in warmer springs. These warblers therefore appeared able to adjust mean lay date appropriately to the 3.3-fold range of variation in spring temperature observed among the 25 years of this demographic study. The fitness responses to warm springs observed for this long-distance migrant were very similar to those documented for a resident population of another North American songbird, the song sparrow [5]. In contrast, several other long-distance migratory species in Europe have experienced increasing selection pressure for early breeding in concert with warming climate trends [12], [13], implying that they were unable to advance their lay date to match warmer springs. Our results suggest that black-throated blue warblers might not experience the same fecundity declines as the European migratory songbirds that are unable to adjust their timing of breeding in pace with springs temperatures.

### Double Brooding, Early Breeding, and Resilience to Climate Change

Breeding initiation date is important in double brooded species because birds that get an early start are generally more likely to produce multiple broods [40]–[42]. Species that produce multiple broods might therefore benefit from a warming climate because of earlier start dates and extended breeding seasons. A recent analysis of 20 bird species in Denmark [19] suggested that the breeding season of double brooders lengthened in warm years, whereas breeding season of single brooders contracted. In one of these double brooding species, the long-distance migratory barn swallow Hirundo rustica, temperature-related extension of its breeding season was linked to a longer interclutch interval, which in turn increased fledging success [18]. In a resident population of song sparrows on Mandarte Island, temperature-linked early breeding had a positive effect on fledging success because females successfully produced more broods when they started earlier [5]. Likewise, in a Polish population of the double brooded reed warbler Acrocephalus scirpaceus, the percentage of birds producing second clutches appears to have increased since the 1970s, which the authors attribute to a temperature-linked increase in breeding season duration [43]. In general, double brooded species might be more resilient to climate change than single brooded species, as suggested by the positive effect of double brooding on climate-linked population trends among 71 bird species in France [17]. Double brooding could have thus contributed to the stable population trends of black-throated blue warblers [44]. Although many Nearctic-Neotropical migratory passerines are classified as single brooded [45], the incidence of double brooding is poorly known for most of these species [35]. Better information about this important life-history trait among long-distance migratory birds would shed light on interspecific variation in population responses to changing climate.

Not all double brooded species appear to benefit from warming climate trends, however. In four populations of great tits in the Netherlands, for example, the fitness benefits and frequency of double brooding have declined with increasing spring temperatures, apparently because of increasing asynchrony between offspring food requirements and peak abundance of the caterpillars of a single moth species [20]. Such trophic mismatches might be less likely to occur in species that, like the black-throated blue warbler [30]–[32], [46], exploit a species-rich community of prey, among which a single temporal peak of abundance during the breeding season is improbable [7], [22].

Because earlier breeding initiation date predicted double brooding and annual fecundity in black-throated blue warblers, future trends toward earlier breeding could confer a population-level advantage, as long as sufficient food is available [42],[47]. Annual fecundity in this species is positively linked to yearling recruitment in the subsequent year, as well as to maintaining the size of the breeding population [23], [48], [49]. We note, however, that previous work, conducted over a much shorter time series (1995–2001), suggested that food availability later in the season was a more important predictor of double brooding and annual fecundity than breeding initiation date [42], [47]. Breeding initiation date and food availability were not independent variables, however: birds began breeding earlier on territories with more food [42], potentially masking the effects of lay date on the probability of double brooding. Population projections should therefore consider variation in food availability along with directional changes in lay date.

### Selection for Early Breeding

Black-throated blue warblers, on average, had higher reproductive success when they initiated breeding earlier, and they were under apparent selection pressure to breed earlier in most years of this study. However, the strength of selection on breeding initiation date appeared to fluctuate across years: the correlation between the residuals of the strength of selection on earlier breeding (φ) was −0.54, suggesting that years with relatively strong, negative selection differentials (i.e., strong selection for earlier breeding) were frequently followed by years with relatively weak or even positive selection (Fig. 3A). The reasons for these fluctuations in the strength of selection are unclear, but nonetheless breeding date did not advance over time despite overall selection for earlier breeding. At least three non-exclusive reasons could explain this lack of response to selection for earlier breeding [50]. First, delayed breeding might be a conservative strategy (i.e., “conservative bet-hedging;” [13]) that persists because of the potentially high, but unpredictable, costs of beginning too early, such as nest failure due to late spring storms and unseasonably cold temperatures. Such weather-related nest failures have been observed at Hubbard Brook [30]. Second, birds in poor condition due to non-heritable causes might be unable to breed as early in the season as birds in superior condition, or they might have a later optimal breeding initiation date, leading to a mean first lay date that is later than the population-level optimum [50]. Indeed, yearling males initiated first clutches later than older birds, and indirect evidence indicates that yearlings might be in poor condition relative to older birds at the time of arrival: yearlings arrive later on the breeding grounds at Hubbard Brook [30] and settle on territories of poor quality [29] relative to older birds. Such delayed arrivals have been linked to poor physiological condition on non-breeding grounds in another Neotropical-Nearctic migrant, the American redstart S. ruticilla [26], [27]. Last, selection pressures may differ across this warbler’s broad range [51]. Because natal dispersal in this species is broad [30] and the population is well mixed [51], selection pressure in one area might be muted or balanced by opposing pressures in other areas.

### Inter-annual Weather Variation vs. Climate Trends

We used the substantial inter-annual variation in spring temperature, rather than trend over time, to infer possible responses to climate change, as have numerous other studies [5], [52]–[55]. Our analyses did not reveal a trend towards earlier breeding or an increase in selection pressure from 1986–2010, which is unsurprising because we saw no trend towards warmer springs within this 25-year interval. However, mean spring temperatures did increase significantly over a longer (53-year) time-series at Hubbard Brook (i.e., since the start of collection of weather data in 1957; see also [24]). Such warming climate trends have been linked to advancing spring leaf phenology at Hubbard Brook [25]. With the continued, directional increases in spring temperature that have been projected for New England in the coming decades [56], our results suggest that a future trend towards earlier breeding likely. Selection pressure for earlier breeding could intensify as well, if this migratory warbler is ultimately constrained from continual advancement of breeding initiation date by fixed spring migration cues [10], [12].

Despite the link between variation in spring temperature and earlier breeding, and the link between early breeding and reproductive output, spring temperature was not a direct predictor of double brooding or annual fecundity. A suite of other environmental and ecological factors limit and regulate annual fecundity in this species [57], including insect abundance [47], predators [58], conspecific density [49], understory foliage density [29], precipitation [32], and global climate cycles [48], and any of these could interact with or mask the effect of temperature on annual fecundity. One such interaction involves conspecific density, climate, and insects: the strength of density dependence on warbler fecundity appears to be stronger in El Niño years, which are characterized by low insect abundance, than in La Niña years [59]. Interactive effects of these climatic and ecological factors, as well as events and conditions during migration and wintering, must also be taken into consideration when predicting the effects of climate change on population trends in long-distance migrants. Spring temperatures alone might have little power to predict annual fecundity and population trends in this species.

### Conclusions

Multi-decade studies that examine the fecundity effects of advancing lay date on bird populations are rare, with results that have varied among species studied [4]. Our study suggests that black-throated blue warblers that breed earlier in the season have a fecundity advantage over later breeding birds. However, possible future trends towards warmer springs, advancing lay date, and increasing annual fecundity might not necessarily translate to population growth in this species, because a number of different demographic parameters contribute to population change [5], [60]. Furthermore, warming climate trends could lead to a decline in habitat quality, particularly in prey abundance, over time [61]. An understanding of the indirect effects of climate variation on the abundance and phenology of prey, as well as the long-term effects of climate trends on habitat structure and quality, will be necessary to project the effects of climate change on populations of Nearctic – Neotropical migratory songbirds.

## Supporting Information

Table S1 Fixed effects from a generalized linear mixed model* predicting the probability of double-brooding (0/1) of black-throated blue warblers. Yearling pairs were less likely to attempt a second brood than pairs containing one or two older birds. Spring temperature = mean daily temperature, 15 March–18 May.
(DOCX)
