# Supplementary material for: Warm Springs, Early Lay Dates, and Double Brooding in a North American Migratory Songbird, the Black-Throated Blue Warbler
Source: PLoS One. 2013 Apr 2;8(4):e59467. doi: 10.1371/journal.pone.0059467 (PMC3614938; doi:10.1371/journal.pone.0059467)
Supplement: Table S1 — Fixed effects from a generalized linear mixed model* predicting the probability of double-brooding (0/1) of black-throated blue warblers. Yearling pairs were less likely to attempt a second brood than pairs containing one or two older birds. Spring temperature = mean daily temperature, 15 March–18 May. (DOCX) [file pone.0059467.s001.docx]

**Supporting Information**

**Table S1.** Fixed effects from a generalized linear mixed model* predicting the probability of double-brooding (0/1) of black-throated blue warblers. Yearling pairs were less likely to attempt a second brood than pairs containing one or two older birds. Spring temperature = mean daily temperature, 15 March – 18 May.

| Response variable | Explanatory variable | ß ± SE | *Z* | *p* |
| --- | --- | --- | --- | --- |
| double-brooding | Spring temperature (^o^C) | 0.03 ± 0.20 | 0.2 | 0.86 |
|  | Yearling female^a^ vs. older pair | -0.36 ± 0.39 | -0.9 | 0.36 |
|  | Yearling male^a^ vs. older pair | -0.38 ± 0.38 | -1.0 | 0.32 |
|  | Yearling pair vs. older pair | -1.17 ± 0.38 | -3.1 | 0.002 |

*Year specified as random effect. *N =* 309 pair years; 25 years

^a^Mixed-age pair; yearling male paired to older female or yearling female paired to older male
